# Application of Tensor Decomposition to Gene Expression of Infection of Mouse Hepatitis Virus Can Identify Critical Human Genes and Efffective Drugs for SARS-CoV-2 Infection

**DOI:** 10.1109/JSTSP.2021.3061251

**Published:** 2021-02-23

**Authors:** Y-H. Taguchi, Turki Turki

**Affiliations:** 1 Department of PhysicsChuo University 12741 Tokyo 112-8551 Japan; 2 Department of Computer ScienceKing Abdulaziz University 37848 Jeddah 21589 Saudi Arabia

**Keywords:** COVID-19, feature extraction, gene expression profile, SARS-CoV-2, tensor decomposition, *in silico* drug discovery

## Abstract

To better understand the genes with altered expression caused by infection with the novel coronavirus strain SARS-CoV-2 causing COVID-19 infectious disease, a tensor decomposition (TD)-based unsupervised feature extraction (FE) approach was applied to a gene expression profile dataset of the mouse liver and spleen with experimental infection of mouse hepatitis virus, which is regarded as a suitable model of human coronavirus infection. TD-based unsupervised FE selected 134 altered genes, which were enriched in protein-protein interactions with orf1ab, polyprotein, and 3C-like protease that are well known to play critical roles in coronavirus infection, suggesting that these 134 genes can represent the coronavirus infectious process. We then selected compounds targeting the expression of the 134 selected genes based on a public domain database. The identified drug compounds were mainly related to known antiviral drugs, several of which were also included in those previously screened with an *in silico* method to identify candidate drugs for treating COVID-19.

## I. Introduction

THE current pandemic of COVID-19 caused by infection of the new coronavirus strain SARS-CoV-2 is a severe public health problem that must be resolved as soon as possible. To achieve this goal, it is essential to understand the mechanism by which SARS-CoV-2 successfully invades human cells. Although there are many *in silico* trials for repositioning drugs toward COVID-19 [1]–[3], most of them are to try to find compounds that bind to SARS-CoV-2 proteins with *in silico* method. On the other hand, we previously identified drug candidate compounds using gene expression profiles of diseases [4], [5]. This strategy can be also applicable to COVID-19. Recently, Pfaender *et al.* [6] demonstrated that host lymphocyte antigen 6 (LY6E) complex impairs coronavirus fusion and confers immune control of viral disease. The authors also used a transcriptome approach to evaluate the effect of infection of mouse hepatitis virus (MHV), a natural mouse pathogen that causes hepatitis and encephalomyelitis, which is a well-studied model of coronavirus infection. Although they found many pathways that were disturbed after MHV infection, they did not perform a detailed analysis of the genes with altered expression in response to MHV infection.

In this study, we applied tensor decomposition (TD)-based unsupervised feature extraction (FE) [7] to identify genes with altered expression by MHV infection as a model of coronavirus. We further performed functional enrichment on the selected genes to determine their potential associations with coronavirus infection processes, and screened candidate drug compounds targeting these genes. Overall, this work expands TD formalism by exploring the interpretation of six-dimensional tensors in an infectious disease context. Moreover, we demonstrate a novel application of TD to facilitate the drug discovery process, which can offer a valuable resource for researchers to obtain mechanistic insight for identifying effective drugs for infectious diseases such as COVID-19.

The reason why TD was primary employed to be applied to the present data set that we investigated is because the data set was formatted as a six-mode tensor. Since TD is the most famous method to be used to attack the data set formatted as tensor, it is natural to apply TD to it. Although any other methods might be applicable, they will be tried only when TD fails (and as can be seen in the below, it is not the case in this study; TD can work quite well). In addition to this, our proposed approach, TD based unsupervised FE [7], was known to be applicable to wide range of genomics study. Thus, the purpose of this study is to propose a different tensor formulation dealing with such different tensor data representation and to estimate how well the existing method can work to fulfill a new requirement; Using the experiments of mice infected by virus that is related to SARS-CoV-2, but is not SARS-CoV-S itself, identifying critical human genes that play important roles when SARS-CoV-2 infects human lung. The key point is if we can outperform the previous study where SARS-CoV-2 infected human lung cell line [8]. If we can derive the better results than those using MHV infected mouse data set, it is considered a remarkable achievement (and we could do this as can be seen in the below). It is worth noting that our TD employs “samples + genotypes + tissues + treatments + biological replicates + technical replicates” structure that requires a six-axes tensor representation to identify critical human genes and effective drugs for SARS-CoV-2 infection. Reported results via enrichment analysis show the superiority of our TD, attributed to taking into account the relationships among samples, genotypes, tissues, treatments, biological replicates, and technical replicates at once”

## II. Materials and Methods

### A. Gene Expression Profile Dataset

The gene expression profile was downloaded from the Gene Expression Omnibus (GEO) dataset GSE146074. This dataset comprises the gene expression profiles of the liver and spleen from female mice experimentally infected with MHV or injected with phosphate-buffered saline (PBS) as a control group for comparison. This experiment was performed with mice of two genetic backgrounds, including wild-type (WT) mice and an textit Ly6e-knockout (KO) mutant strain. The number of replicates for each group are listed in Table I. Seventy-two files whose names start with “GSM” (processed file) were used for the analyses.

### B. Additional Gene Expression Profile Datasets

In order to validate the suitability of MHV as model SARS-CoV-2 infectious process, two additional gene expression profiles of mouse lung SARS-CoV infectious processes were used (Table II).

**TABLE I:** Number of Biological Replicates. Two Technical Replicates are Available for Each Biological Replicate

	PBS day 5		MHV day 3		MHV day 5	
	liver	spleen	liver	spleen	liver	spleen
WT	3	3	3	3	3	3
KO	3	3	3	3	3	3

**TABLE II:** Number of Biological Replicates of SARS-CoV Infection Toward Mouse Lung Gene Expression Profiles

GSE33266				
days	D1	D2	D4	D7
Mock	3	3	3	3
$10^2$pfu	5	5	5	5
$10^3$pfu	5	5	5	5
$10^4$pfu	5	5	5	5
$10^5$pfu	5	5	5	5
GSE50000				
days	d1	d2	d4	d7
BatSRBD	5	5	5	4
icSARS	4	5	5	5
Mock	4	4	4	4

For GSE33266 and GSE50000, we downloaded two files: GSE33266_series_matrix.txt.gz and GSE50000_series_matrix.txt.gz. Although GSE50000 also includes files for SARS-CoV-MA15, they were omitted for reasons detailed in the Discussion section. For cases with less than five biological replicates, we used some replicates more than once, in order to have five biological replicates for individual cases.

### C. TD-Based Unsupervised FE

Although the TD-based unsupervised FE was fully described in the recent book [7], we briefly outline the analysis flow. At first, TD is applied to tensor and singular value vectors are obtained. Individual singular value vectors are attributed to either various experiments or genes. By investigating singular value vectors attributed to experiments, we identify which ones are associated with properties of interest. Then among singular value vectors attributed to genes, those coincident with identified singular value vectors attributed to experiments are selected. Finally, genes having larger contributions toward selected singular value vectors attributed to genes are selected as those associated with the properties of interest.

**Fig. 1.**
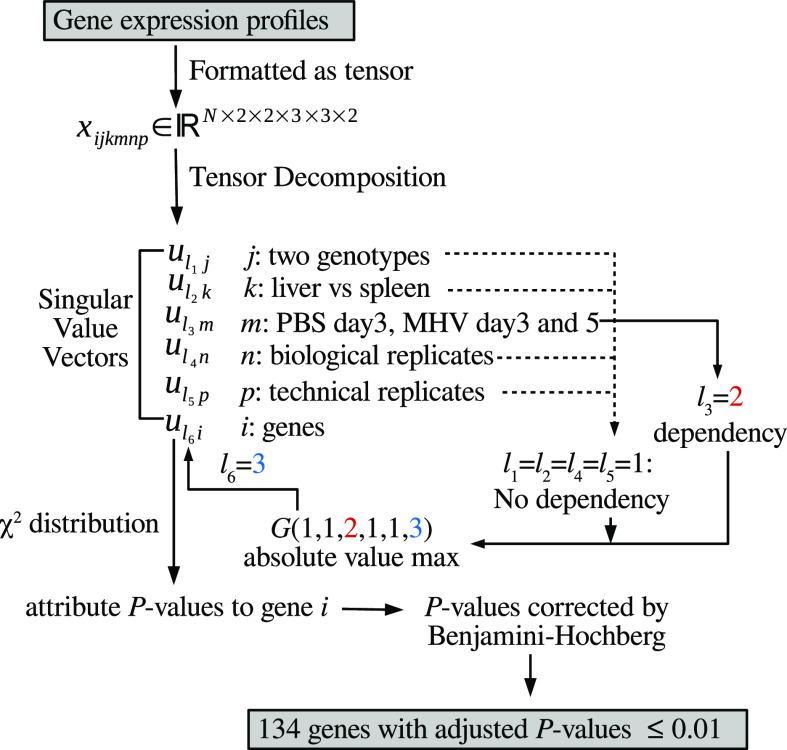
Flowchart of TD-based unsupervised FE. No dependency: independent of $j,k,n,p$, dependency: dependent upon $m$. Singular value vectors with $\ell _1=\ell _2=\ell _5=\ell _5=1, \ell _3=2$ are selected.

Fig. 1 shows the flowchart of TD-based unsupervised FE.

The gene expression profile dataset was formatted as a tensor, $x_{ijkmnp} \in \mathbb {R}^{N \times 2 \times 2 \times 3 \times 3 \times 2}$, which represents the expression level of the $i$th gene of the $j$th genotype ($j=1$:KO, $j=2$:WT) of the $k$th tissue ($k=1$:liver, $k=2$:spleen) of the $m$th treatment group ($m=1$:PBS day 5, $m=2$:MHV day 3, $m=3$:MHV day 5) for the $n$th biological replicate ($1 \leq n \leq 3$) and $p$th technical replicate ($1 \leq p \leq 2$).

The TD is therefore expressed as

\begin{align*}
x_{ijkmnp} = & \sum _{\ell _1 \ell _2 \ell _3 \ell _4 \ell _5 \ell _6} G(\ell _1 \ell _2 \ell _3 \ell _4 \ell _5 \ell _6) \\
&\times u_{\ell _1 j} u_{\ell _2\,k} u_{\ell _3\,m} u_{\ell _4 n} u_{\ell _5 p} u_{\ell _6 i} \tag{1}
\end{align*}

where $G(\ell _1 \ell _2 \ell _3 \ell _4 \ell _5 \ell _6) \in \mathbb {R}^{ 2 \times 2 \times 3 \times 3 \times 2 \times N }$ is a core tensor, and $u_{\ell _1 j} \in \mathbb {R}^{2 \times 2}$, $u_{\ell _2\,k} \in \mathbb {R}^{2 \times 2}$, $u_{\ell _3\,m} \in \mathbb {R}^{3 \times 3}$, $u_{\ell _4 n} \in \mathbb {R}^{3 \times 3}$, $u_{\ell _5 p} \in \mathbb {R}^{2 \times 2}$, and $u_{\ell _6 i} \in \mathbb {R}^{N \times N}$ are singular values vectors, which can be obtained via the higher-order singular value decomposition (HOSVD) algorithm [7].

To select $u_{\ell _6i}$, attributed to selected genes, we need to select $u_{\ell _1 j}$ attributed to the genotype, $u_{\ell _2\,k}$ attributed to the tissue, $u_{\ell _3\,m}$ attributed to the treatment, $u_{\ell _4 n}$ attributed to the biological replicate, and $u_{\ell _5 p}$ attributed to the technical replicate, associated with desired properties.

For this study, we sought to identify genes whose expression is independent of the mouse genotype, tissue type, and replicate. Thus, $u_{\ell _1 j}$, $u_{\ell _2\,k}$, $u_{\ell _4 n}$, and $u_{\ell _5 p}$ should be independent of $j$, $k$, $n$, and $p$.

By contrast, we require $u_{\ell _3\,m}$ to be dependent on $u_{\ell _3 1} < u_{\ell _3 2} < u_{\ell _3 3}$ or vice versa. This is because $m=2$ (3 days after MHV infection) must be between $m=1$ (5 days after PBS injection as the control) and $m=3$ (5 days after MHV infection).

After selecting $\ell _1, \ell _2, \ell _3$, $\ell _4$, and $\ell _5$ based on the above considerations, we selected $\ell _6$ associated with $G(\ell _1 \ell _2 \ell _3 \ell _4 \ell _5 \ell _6)$ as the largest absolute value, with fixed $\ell _1, \ell _2, \ell _3, \ell _4$, and $\ell _5$ values. Using the selected $u_{\ell _6 i}$, The $P$-values, $P_i$s, were attributed to gene expression levels as

\begin{equation*}
P_i = P_{\chi ^2} \left[ > \left(\frac{u_{\ell _6 i}}{\sigma _{\ell _6}}\right)^2 \right] \tag{2}
\end{equation*}

where $P_{\chi ^2}[>x]$ is the cumulative probability distribution of the $\chi ^2$ distribution when the argument is larger than $x$ and $\sigma _{\ell _6}$ is the standard deviation of $u_{\ell _6 i}$.

$P_i$s were adjusted by the Benjamini and Hochberg (BH) criterion [7], and only genes associated with an adjusted $P_i$ less than 0.01 were selected for further analysis.

We employed almost the same procedures, apart from different tensor fromats for the two additional gene expression profiles of mouse lung SARS-CoV infectious processes. The tensors formatted and TDs are for GSE33266

\begin{align*}
x_{ijkn} \in & \mathbb {R}^{N \times 5 \times 4 \times 5} \tag{3}
\\
= & \sum _{\ell _1}^5 \sum _{\ell _2=1}^4 \sum _{\ell _3=1}^5 \sum _{\ell _4=1}^N G(\ell _1 \ell _2 \ell _3 \ell _4) \\
&\times u_{\ell _1j} u_{\ell _2\,k}u_{\ell _3n}u_{\ell _4i} \tag{4}
\end{align*}

which represents the $i$th gene expression profile of $j$th experiments ($j=1$:Mock, $j=2$:$10^2$pfu, $j=3$:$10^3$pfu, $j=4$:$10^4$pfu, $j=5$:$10^5$pfu) at the $k$th day after infection ($k=1$:D1, day 1, $k=2$:D2, day 2, $k=3$:D4, day 4, $k=4$:D7, day 7) of the $n$th biological replicate ($1 \leq n \leq 5$). $G(\ell _1 \ell _2 \ell _3 \ell _4) \in \mathbb {R}^{ 5 \times 4 \times 5 \times N}$ is a core tensor that represents the weight of products of the singular value matrices $u_{\ell _1 j} \in \mathbb {R}^{5 \times 5}$, $u_{\ell _2\,k} \in \mathbb {R}^{4 \times 4}$, $u_{\ell _3 n} \in \mathbb {R}^{5 \times 5}$, and $u_{\ell _4 i} \in \mathbb {R}^{N \times N}$, which are all orthogonal matrices. Those for GSE50000 are

\begin{align*}
x_{ijkn} \in & \mathbb {R}^{N \times 3 \times 4 \times 5} \tag{5}\\
= & \sum _{\ell _1}^3 \sum _{\ell _2=1}^4 \sum _{\ell _3=1}^5 \sum _{\ell _4=1}^N G(\ell _1 \ell _2 \ell _3 \ell _4) \\
&\times u_{\ell _1j} u_{\ell _2\,k}u_{\ell _3n}u_{\ell _4i} \tag{6}
\end{align*}

which represents the $i$th gene expression profile of the $j$th experiments ($j=1$:BatSRBD, $j=2$:icSARS, $j=3$:Mock) at the $k$th day after infection ($k=1$:d1, day 1, $k=2$:d2, day 2, $k=3$:d4, day 4, $k=4$:d7, day 7) of the $n$th biological replicate ($1 \leq n \leq 5$). $G(\ell _1 \ell _2 \ell _3 \ell _4) \in \mathbb {R}^{ 3 \times 4 \times 5 \times N }$ is a core tensor that represents the weight of products of singular value matrices, $u_{\ell _1 j} \in \mathbb {R}^{3 \times 3}$, $u_{\ell _2\,k} \in \mathbb {R}^{4 \times 4}$, $u_{\ell _3 n} \in \mathbb {R}^{5 \times 5}$, and $u_{\ell _4 i} \in \mathbb {R}^{N \times N}$ which are all orthogonal matrices.

The criteria for the selection of singular value vectors were as follows. For GSE33266, $u_{\ell _1 j}$ should be a monotonic function of $j$, since it represents the strength of infection, $u_{\ell _2\,k}$ should also be a monotonic function of $k$, since it represents time development, and $u_{\ell _3 n}$ should be constant, since biological replicates should not differ from one another. For GSE50000, $u_{\ell _1 j}$ should be distinct between $k=3$ and $k=1,2$, since it represents the distinction between mock and real infection, $u_{\ell _2\,k}$ also should be a monotonic function of $k$, since it represents time development, and $u_{\ell _3 n}$ should be constant, since biological replicates should not differ from one another.

Downstream procedures for gene selection after identifying singular value vectors are the same as those for MHV. The core tensors, $G(\ell _1 \ell _2 \ell _3 \ell _4)$s, were investigated in order to see which $\ell _4$ associated with $G(\ell _1 \ell _2 \ell _3 \ell _4)$ having larger absolute values given $\ell _1, \ell _2, \ell _3$. $u_{\ell _4 i}$s selected are used for attributing $P$-values, $P_i$, to genes. Genes associated with adjusted $P$-values less than 0.01 were selected.

### D. Enrichment Analysis

Gene symbols of genes selected by TD-based unsupervised FE with significantly altered expression due to MHV infection were uploaded to Enricher [9] and Metascape [10], which are popular enrichment analysis servers that evaluate the biological properties of genes based on enrichment analysis. There are some explanations about individual categories of Enrichr.

#### 1) Virus-Host PPI P-HIPSTer2020

This is a list of human proteins known to interact with various virus proteins. By comparing uploaded genes with genes in the list, we can estimate the ratio of genes interacting with virus proteins.

#### 2) Virus Perturbations From GEO up/down

From GEO, gene expression profiles of virus infections are retrieved. Then genes associated with altered expression are identified. By comparing uploaded genes with genes in the list, we can estimate the ratio of genes whose expression is altered by virus infection.

#### 3) Drugmatrix

In DrugMatrix data base, gene expression profiles of rat tissues treated with various drugs are recorded. By comparing uploaded genes with genes in the list, we can estimate the ratio of genes whose expression is altered by drug treatment.

#### 4) Drug Perturbations From GEO up/down

From GEO, gene expression profiles of drug treatments are retrieved. Then genes associated with altered expression are identified. By comparing uploaded genes with genes in the list, we can estimate the ratio of genes whose expression is altered by drug treatment.

## III. Results

**Fig. 2.**
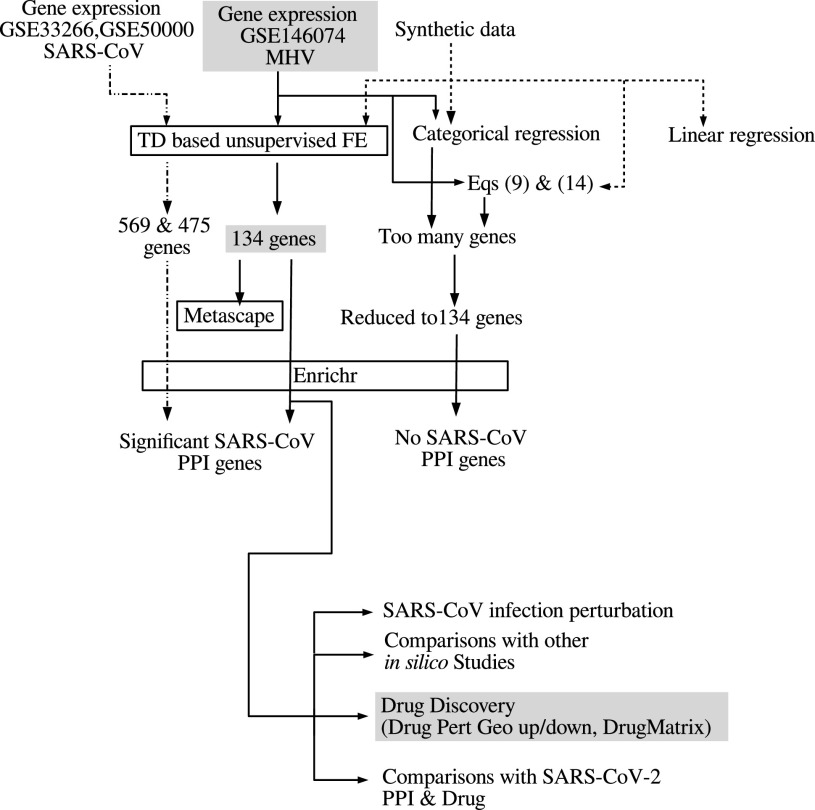
Overview of analyses.

Fig. 2 shows the overview of analyses performed in this study.

### A. Synthetic Data Sets

In order to demonstrate how effective the tensor is, we employed synthetic data sets composed of multiway and multiclass labels, $x_{ijks} \in \mathbb {R}^{N \times M \times K \times S}$. Here, $i$ represents $N$ variables, among which partial collections having distinct values between $j$s as well as $k$s must be selected, where $S$ replicates are available for individual combinations of $j$ and $k$. A typical example is that $x_{ijks}$ represents the expression of the $i$th gene in the $k$th tissue of patients who belong to the $j$th group, that consists of $S$ patients. We then need to identify which genes are expressed distinctly in tissue ($k$) -specific as well as patient-group ($j$) specific ways.

The most popular approach to this problem is linear regression,

\begin{equation*}
x_{ijks} = \alpha _i a_j +\beta _i b_k + \gamma _i \tag{7}
\end{equation*}

where $a_j$ and $b_k$ are pre-defined variables that represent some properties of the $j$th patient group and $k$th tissue, respectively. $\alpha _i, \beta _i$, and $\gamma _i$ are regression coefficients that are selected such that the discrepancy between both sides of equation is minimized. Then the $i$s associated with significant $P$-values are selected as those whose expression levels are different between distinct $j$s and $k$s.

It is not guaranteed that liner regression will correctly represent the dependency of $x_{ijks}$ upon $j$ and $k$. We generated synthetic data that follows

\begin{equation*}
x_{ijks} = \xi _{ijk} a_jb_k + \varepsilon _{ijks} \tag{8}
\end{equation*}

where $\xi _{ijk}$ are drawn from ${\mathcal N}(1,0)$ for every combination of $i,j,k$, so that $x_{ijks}$s associated with distinct $s$s share the same $\xi _{ijk}$, and $\varepsilon _{ijks}$ are drawn from ${\mathcal N}(1,0)$ for every combination of $i,j,k,s$. ${\mathcal N}(\mu, \sigma)$ represents a normal distribution that has mean of $\mu$ and standard deviation of $\sigma$. For $i > N_0$, $\xi _{ijk}$ is taken to be zero; this means that $x_{ijks}$ for $i > N_0$ is simply random variables. The task is to correctly select $N_0$ variables associated with the dependence upon $j$ and $k$.

As an alternative regression analysis to linear regression, we employed

\begin{equation*}
x_{ijk} = \alpha ^{\prime }_i a_j b_k + \gamma ^{\prime }_i \tag{9}
\end{equation*}

which reflects the multiplicative nature when $x_{ijk}$ is generated with eq. (8) although it cannot represent $x_{ijk}$ completely since $\xi _{ijk}$ is replaced with $\alpha ^{\prime }_i$ and therefore dependence upon $j$ or $k$ is not assumed.

In addition to the above two regression analyses, we applied categorical regression, which is equivalent to analysis of variance (ANOVA):

\begin{equation*}
x_{ijks} = \alpha _{ijk} + \gamma ^{\prime \prime }_i \tag{10}
\end{equation*}

where $\alpha _{ijk}$ and $\gamma ^{\prime \prime }_i$ are regression coefficients. Eq. (10) can fully reproduce the $x_{ijk}$ generated by eq. (8), when $\alpha _{ijk}$ is taken to be $\xi _{ijk}a_{j}b_{k}$ excluding randomness introduced by $\varepsilon _{ijks}$, which can be regarded as residuals.

Finally, we also applied TD based unsupervised FE to $x_{ijks}$. TD is computed as

\begin{equation*}
x_{ijks} = \sum _{\ell _4=1}^N \sum _{\ell _1=1}^M \sum _{\ell _2=1}^K \sum _{\ell _3=1}^S G(\ell _1 \ell _2 \ell _3 \ell _4) u_{\ell _1 j} u_{\ell _2\,k} u_{\ell _3\,s} u_{\ell _4 i} \tag{11}
\end{equation*}

Then $\ell _1$ and $\ell _2$, that have the largest absolute values of the correlation coefficient between $u_{\ell _1 j}$ and $a_j$ or $u_{\ell _2\,k}$ and $b_k$ were selected. The top two $\ell _1$ that have the largest absolute $G(\ell _1 \ell _2 1 \ell _4)$s were selected. The reason why $\ell _3$ is fixed to be 1 is because $u_{\text{1}\,s}$ always represent the $u_{\ell _3\,s}$ that lacks $s$ dependency, i.e., is a constant independent of $s$. Since $x_{ijks}$ should take the same values between replicates, $u_{\ell _3\,s}$ that do not have any $s$ dependence are selected. Then $P$-values are attributed to $i$ as

\begin{equation*}
P_i = P_{\chi ^2} \left[ > \sum _{\ell _4}\left(\frac{u_{\ell _4 i}}{\sigma _{\ell _4}}\right)^2\right] \tag{12}
\end{equation*}

where summation is taken over two selected $\ell _4$ only.

No matter which form of linear regression, eq.(7), eq. (9), categorical regression, eq.(10), or TD, eq.(11), was used to compute $P_i$, $P_i$ was corrected using the BH criterion [7] and $i$s having corrected $P_i$ less than threshold $P$-values were selected as those associated with dependency upon $j$ and $k$, respectively.

For simplicity, we employed $a_j=j$ and $b_k=k$ when generating $x_{ijks}$ using eq. (8) as well as eq. (9) for regression analysis. Specifically, we chose $N=1000, N_0=100, M=K=3, S=5$. Generation of $x_{ijks}$ and selection of $i$s were repeated 100 times with two distinct threshold $P$-values, 0.01 or 0.1, for each trial. Table III shows the performance averaged over 100 trials. Although categorical regression, eq. (10), could outperform the other three approaches, since it is able to completely reproduce eq. (8) as shown above, it has one weak point: it cannot explicitly consider the dependence of $a_j$ and $b_k$ upon $j$ and $k$, since $\xi _{ijk}a_jb_k$s are estimated as one parameter, $\alpha _{ijk}$. This limitation might prevent us from selecting $i$s that are specifically associated with the dependence upon $j$ and $k$ that $a_j$ and $b_k$ represent. Even if some $i$s are selected, it might be because of dependence upon $j$ and $k$ that $a_j$ and $b_k$ do not represent. There is no way for us to check this point. Taking this problem into account, TD based unsupervised FE, which is the second best approach, is more useful than categorical regression, since TD based unsupervised FE can consider $a_j$ and $b_k$ when selecting $u_{\ell _1j}$ and $u_{\ell _2\,k}$ correlated with $a_j$ and $b_k$.

**TABLE III:** The Confusion Matrices Obtained Using Synthetic Data (eq.(8), $N=1000, N_0=100, M=K=3, S=5$) and Cpu Time Required for Each Method. $P$s are Threshold $P$-Values; $i$ Associated With Adjusted $P$-Vales Less Than This Threshold Values are Selected

$P$	0.01		0.1	
TD based unsupervised FE (eq. (11), cpu time 6.5 sec)				
	not selected	selected	not selected	selected
$i>N_0$	900	62.2	899.9	48.44
$i\leq N_0$	0	37.8	0.1	51.56
liner regression (eq. (7), cpu time 65.7 sec)				
	not selected	selected	not selected	selected
$i>N_0$	899.7	75.39	894.0	58.31
$i\leq N_0$	0.3	24.61	6.0	41.69
categorical regression (cpu time 70.4 sec)				
	not selected	selected	not selected	selected
$i>N_0$	897.3	0	—	—
$i\leq N_0$	2.7	100	—	—
eq. (9) (cpu time 84.1 sec)				
	not selected	selected	not selected	selected
$i>N_0$	900	84.55	899.3	74.0
$i\leq N_0$	0	14.55	0.7	26.0

Another advantage of TD based unsupervised FE is the short CPU time required for execution (Table III). The CPU time required for TD based unsupervised FE is approximately one tenth that of other three methods, which must repeat the regression analysis $N$ times. This difference might be important when dealing with massive data sets.

TD based unsupervised FE can consider the dependence upon $j$ and $k$ of $a_j$ and $b_k$, although other methods cannot because of the random nature of $\xi _{ijk}$ in eq. (8). When an individual $i$ is considered, there are no ways to take into account the dependence upon $j$ and $k$ that $a_j$ and $b_k$ have. However, when $x_{ijks}$s are averaged over multiple $i$s, there is a possibility that the dependence upon $j$ and $k$ of $a_j$ and $b_k$ can appear, since the randomness of $\xi _{ijk}$ can be smeared out because of averaging. In practice, $u_{\ell _1j}$ and $u_{\ell _2\,k}$ can be such variables that can appear only after averaging, and can represent the dependence upon $j$ and $k$ of $a_j$ and $b_k$. This is why TD based unsupervised FE can outperform eq. (9), which explicitly considers the multiplicative nature when $x_{ijks}$s are generated, and can consider the dependence upon $j$ and $k$ of $a_j$ and $b_k$, which categorical regression, eq.(10), cannot.

### B. Selection of Genes

We applied TD based unsupervised FE to the gene expression profiles introduced in the Materials and Methods section. Then other methods were applied to the synthetic data set to form a basis for the comparisons discussed in the Discussions and Conclusions section.

**Fig. 3.**
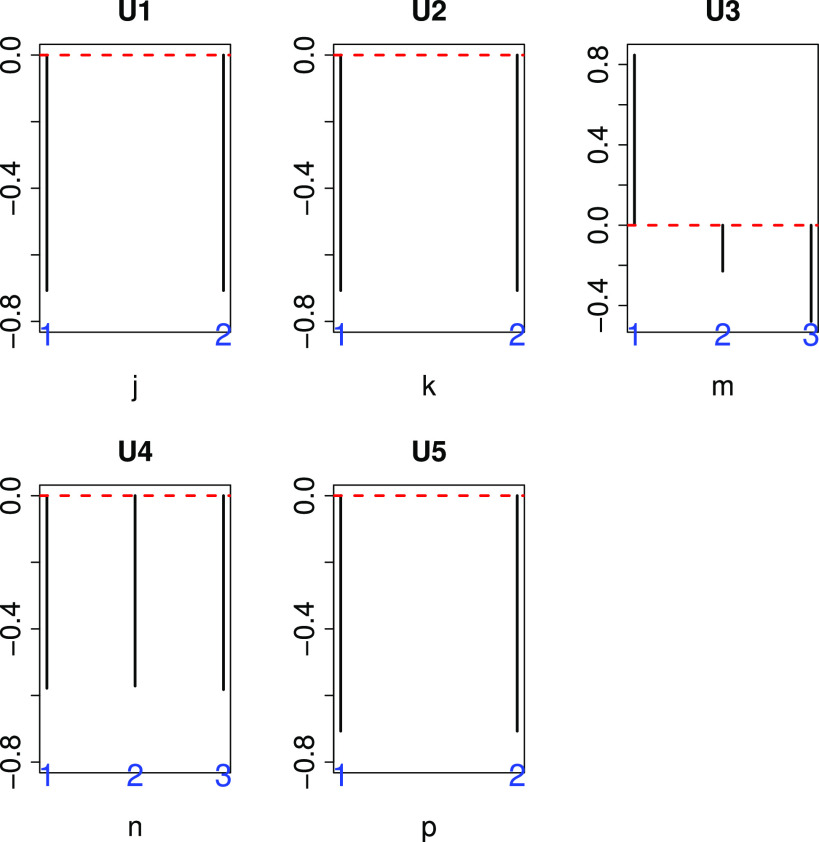
Singular value vectors obtained by the HOSVD algorithm. U1:$u_{1j}$, U2:$u_{\text{1}\,k}$, U3:$u_{\text{2}\,m}$, U4:$u_{\text{1}\,n}$, and U5:$u_{1p}$. See Materials and Methods for the meanings of $j, k, m, n$, and $p$. They are independent of genotypes (j), tissues (k), biological replicates (n) and technical replicates (p), but dependent upon infection (m).

We selected $\ell _1=1, \ell _2=1, \ell _3=2, \ell _4=1$, and $\ell _5=1$ based on the criteria described above (Fig. 3), and the associated $G(1,1,2,1,1,\ell _6)$ values are listed in Table IV, demonstrating the largest value for $G(1,1,2,1,1,3)$. The associated $P_i$ values were computed using $u_{3i}$ as shown in eq. (2), resulting in selection of 134 genes altered in MHV infection with adjusted P-values less than 0.01 (Table V). Although some mouse-specific genes were included in this list (e.g., genes with symbols starting with “mt”), since there were still several gene symbols that are common between human and mice, we decided to evaluate the potential association of all 134 genes with the infection process of coronavirus.

In order to see if we could correctly select differentially expressed genes between infected mice and control, we applied *t* test to expression of 134 genes between infected and control mouse. Then we have found that the null hypothesis that gene expression averaged over selected 134 genes are equal between infected and control mouse was rejected by the $P$-values of 0.004. Thus we could successfully identify expressed genes between infected mice and control.

**TABLE IV:** $G(1,1,2,1,1,\ell _6)$s Computed by the HOSVD Algorithm

$\ell _6$	$G(1,1,2,1,1,\ell _6)$	$\ell _6$	$G(1,1,2,1,1,\ell _6)$
1	-11.846 381	6	22.375 546
2	-28.104 674	7	-41.997 092
3	312.362 569	8	-9.048 416
4	-71.001 444	9	9.212 773
5	-189.719 321	10	3.394 629

**TABLE V:** One Hundred and Thirty Four Genes Selected by TD-Based Unsupervised FE

Actb Actg1 Ahsg Alb Ambp Apoa1 Apoa2 Apoc1 Apoe B2m Bst2 C3 Ccnb1ip1 Cd74 Cfb Eef1a1 Eef1g Eef2 Fabp1 Fau Fga Fgb Fgg Fth1 Ftl1 Gapdh Gc Gm10800 Gm2000 Gpx1 H2-Aa H2-D1 H2-K1 H2-T23 Hamp Hba-a2 Hbb-bs Hbb-bt Hist1h3b Hist1h4h Hist2h2aa2 Hp Hpx Hsp90ab1 Hsp90b1 Hspa8 Ifi27l2a Ifitm3 Lars2 Lcn2 Lyz2 mt-Atp6 mt-Atp8 mt-Co1 mt-Co2 mt-Co3 mt-Cytb mt-Nd1 mt-Nd2 mt-Nd3 mt-Nd4 Mt1 Mt2 Myh9 Orm1 Orm2 Pabpc1 Psap Ptma Rack1 Rpl10-ps3 Rpl11 Rpl12 Rpl13 Rpl13a Rpl14 Rpl17 Rpl19 Rpl23a Rpl26 Rpl3 Rpl32 Rpl36 Rpl36a Rpl37a Rpl38 Rpl4 Rpl41 Rpl5 Rpl6 Rpl7 Rpl7a Rpl8 Rplp0 Rplp1 Rplp2 Rps11 Rps12 Rps14 Rps15 Rps17 Rps18 Rps2 Rps21 Rps23 Rps24 Rps27a Rps27rt Rps29 Rps3 Rps3a1 Rps4x Rps5 Rps6 Rps7 Rps8 Rps9 Rpsa S100a8 S100a9 Saa1 Saa2 Serpina1a Serpina1b Serpina1c Serpina1d Serpina3k Tmsb4x Tpt1 Trf Ttr Ubb Ubc Wfdc21

### C. Protein-Protein Interaction With Coronavirus Infection

We first evaluated whether the 134 selected genes could reflect the process of coronavirus infection using the Enrichr server for functional enrichment analysis. Several of the genes were enriched in the category “Virus-Host PPI P-HIPSTer 2020,” which is related to SARS-CoV (Table VI, see the supplementary materials for the full list).

**TABLE VI:** SARS-CoV-Related Virus PPI in Enrichr

Term	Overlap	P-value	Adjusted P-value
SARS coronavirus excised_polyprotein 1..4369 (gene: orf1ab)	10/194	$7.52 \times 10^{-7}$	$1.68 \times 10^{-3}$
SARS coronavirus P2 full_polyprotein 1..4382	10/198	$9.06 \times 10^{-7}$	$1.01 \times 10^{-3}$
SARS coronavirus nsp7-pp1a/pp1ab (gene: orf1ab)	4/36	$9.61 \times 10^{-5}$	$2.08 \times 10^{-2}$
SARS coronavirus 3C-like protease (gene: orf1ab)	3/19	$2.63 \times 10^{-4}$	$3.05 \times 10^{-2}$

These genes were also related to ORF1ab, polyprotein, and 3C-like protease. Interestingly, Woo *et al.* [11] suggested that ORF1ab, which encodes a replicase polyprotein of CoV-HKU1, is cleaved by papain-like proteases and 3C-like proteinase. Thus, it is reasonable that ORF1ab, polyprotein, and 3C-like protease would be affected during MHV infection VI. Other PPIs detected that are not listed in Table VI (see supplementary materials) were also mainly associated with ORF1ab and polyproteins, suggesting that our strategy has clear capability to elucidate the basic infectious process at the molecular level that is common among various coronaviruses.

### D. Virus Perturbation

We next evaluated whether genes with known altered expression by virus perturbation overlapped with the 134 genes selected by our TD-based unsupervised FE approach (Table VII; see the supplementary material for the full list).

**TABLE VII:** Virus Perturbation in Enricher

Term	Overlap	P-value	Adjusted P-value
up			
SARS-ddORF6 24Hour GSE47961	10/300	$3.52 \times 10^{-5}$	$3.79 \times 10^{-3}$
cSARS Bat SRBD 60Hour GSE37827	9/300	$1.93 \times 10^{-4}$	$1.04 \times 10^{-2}$
SARS-BatSRBD 60Hour GSE47961	8/300	$9.51 \times 10^{-4}$	$4.38 \times 10^{-2}$
down			
SARS-BatSRBD 96Hour GSE47960	11/300	$5.77 \times 10^{-6}$	$4.66 \times 10^{-4}$
SARS-BatSRBD 84Hour GSE47960	9/300	$1.93 \times 10^{-4}$	$6.24 \times 10^{-3}$
cSARS Bat SRBD 60Hour GSE37827	8/300	$9.51 \times 10^{-4}$	$2.36 \times 10^{-2}$

Among these, we detected the overlap of many genes that are pertubated in response to either SARS-CoV or SARS-like bat CoV, which are the genetically closest coronaviruses to the new SARS-CoV-2 strain. This further suggests that our results could have high similarity to the genes perturbated in SARS-CoV-2 infection.

### E. TMPRSS2 as a Scavenger Receptor

**Fig. 4.**
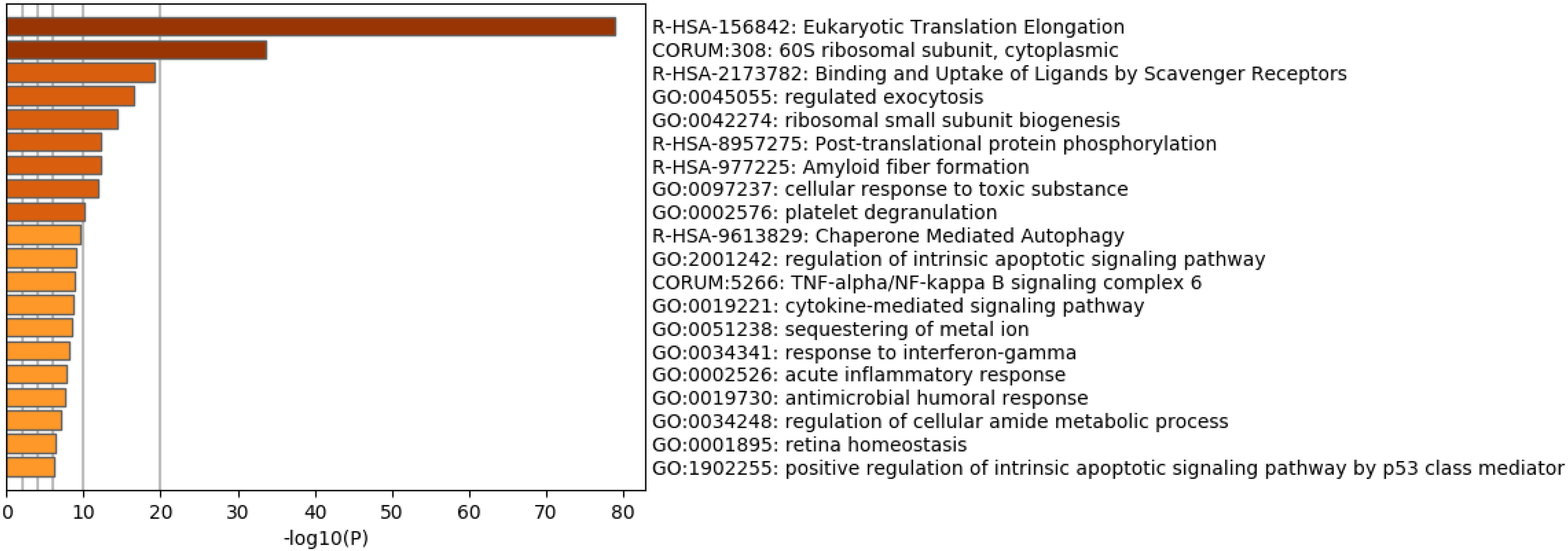
Redundant heatmap of enriched terms generated by uploading the selected 134 genes to Metascape .

For further functional enrichment analysis, we uploaded the 134 selected genes to Metascape to identify non-redundant biological terms (Fig. 4). Among the terms identified, “R-HSA-2 173 782: Binding and Uptake of Ligands by Scavenger Receptors” was the third most significantly enriched term. Although it was initially surprising that a scavenger receptor might be related to the response to coronavirus infection, a search of the related literature revealed that the scavenger receptor TMPRSS2 plays a critical role in SARS-CoV-2 infection as well as SARS-CoV infection [12]. Isolation of SARS-CoV-2 was also reported to be enhanced by TMPRSS2-expressing cells  [13]. Moreover, TMPRSS2 contains a scavenger receptor domain [14], suggesting that TMPRSS2 activity would be related to detection of scavenger receptor activity. This finding further demonstrates the outstanding capability of our strategy to detect factors related to the SARS-CoV-2 infectious process. Moreover, this analysis suggests that research on the SARS-CoV infection process could be informative for understanding the SARS-CoV-2 infection process when it is not possible to directly investigate SARS-CoV-2 infection.

### F. Drug Discovery

We previously demonstrated that genes selected by TD-based unsupervised FE are useful to screen for drugs that are effective in treating disease or those that may cause adverse effects [15]. Therefore, we used this approach to screen for candidate drugs to treat coronavirus infections based on the individual terms that emerged from the Enrichr analysis.

#### 1) Drug Matrix

In the Enrichr category “DrugMatrix,” the top-ranked drug was related to virus infection (Table VIII; see the supplementary materials for the full list). Most of these viruses are enveloped, single-stranded RNA viruses. Coronaviruses, including SARS-CoV-2, are positive-sense, enveloped, single-stranded RNA viruses, whereas influenza virus is a negative-sense, enveloped, single-stranded RNA virus.

**TABLE VIII:** Drugs Enriched in the “DrugMatrix” Category in Enrichr. the Full List is Available in the Supplementary Material

Term	Overlap	P-value	Adjusted P-value
Primaquine-45 mg/kg in CMC-Rat-Liver-5d-up	23/315	$1.40\times 10^{-17}$	$1.10\times 10^{-13}$
Meloxicam-33 mg/kg in Corn Oil-Rat-Kidney-1d-up	23/337	$6.19\times 10^{-17}$	$2.44\times 10^{-13}$
Cytarabine-487 mg/kg in Saline-Rat-Liver-0.25d-up	22/300	$6.87\times 10^{-17}$	$1.80\times 10^{-13}$
Clotrimazole-60 uM in DMSO-Rat-Primary rat hepatocytes-0.67d-dn	24/381	$7.59\times 10^{-17}$	$1.49\times 10^{-13}$
Diclofenac-3.5 mg/kg in Corn Oil-Rat-Liver-5d-dn	21/269	$1.04\times 10^{-16}$	$1.64\times 10^{-13}$
Pyrogallol-1000 mg/kg in Water-Rat-Liver-3d-up	23/349	$1.33\times 10^{-16}$	$1.75\times 10^{-13}$
Clindamycin-161 mg/kg in Saline-Rat-Kidney-1d-up	23/366	$3.76\times 10^{-16}$	$4.23\times 10^{-13}$
Catechol-195 mg/kg in Saline-Rat-Liver-0.25d-up	21/290	$4.76\times 10^{-16}$	$4.69\times 10^{-13}$
Anisindione-75 mg/kg in CMC-Rat-Liver-5d-up	21/295	$6.72\times 10^{-16}$	$5.88\times 10^{-13}$
Phenylhydrazine-78 mg/kg in Water-Rat-Liver-3d-up	22/335	$7.00\times 10^{-16}$	$5.51\times 10^{-13}$
N-Nitrosodiethylamine-1.67 mg/kg in Saline-Rat-Liver-0.25d-up	23/377	$7.15\times 10^{-16}$	$5.12\times 10^{-13}$
Neomycin-56 mg/kg in Corn Oil-Rat-Liver-1d-dn	20/259	$7.28\times 10^{-16}$	$4.78\times 10^{-13}$

Primaquine is known to inhibit the replication of Newcastle disease virus  [16], which is in the family of paramyxoviruses that are enveloped, non-segmented, negative-sense single-stranded RNA viruses. Meloxicam is known to have cytotoxic and antiproliferative activity on virus-transformed tumor cells [17], including myelocytomatosis virus and Rous sarcoma virus. Myelocytomatosis virus is a retrovirus, which is an enveloped, negative-sense, single-stranded RNA virus, whereas Rous sarcoma virus is an enveloped, positive-sense, single-stranded RNA virus. Although there are no studies showing that cytarabine is effective against infection of an RNA virus, one report demonstrated that cytarabine can affect DNA virus infection [18]. Pyrogallol was reported to have anti-virus effects on human influenza virus strain A/Udorn/72, avian influenza virus A/swan/Shimane/499/83, herpes simplex virus-1, vesicular stomatitis virus, and retrovirus [19]. As mentioned above, influenza virus is a negative-sense, enveloped, single-stranded RNA virus; herpesvirus is a DNA virus; vesicular stomatitis virus is an enveloped, single-stranded, negative-sense RNA virus; and retroviruses are enveloped, negative-sense, single-stranded RNA viruses. This suggests that a single drug can effectively inhibit a wide range of viruses from DNA viruses to both negative- and positive-sense RNA viruses. The structure-dependent antiviral activity of catechol derivatives in pyroligneous acid against encephalomyocarditis virus was reported, which is a non-enveloped single-stranded RNA virus [20]. To our knowledge, there are no reports that neomycin is effective against RNA viruses; however, one study showed that it could inhibit infection of fibroblasts with human cytomegalovirus  [21], which is a DNA virus.

Although not all viruses identified to be related to the 134 genes selected by TD-based unsupervised FE are enveloped, positive-sense, single-stranded RNA viruses similar to SARS-CoV-2, since drugs shown to be effective against other viruses (e.g., DNA viruses) are also often effective against RNA viruses (including pyrogallol that was screened by our strategy), drugs in Table VIII warrant being tested as potential treatments for SARS-CoV-2 infection.

#### 2) Drug Perturbations From GEO

Several promising drug compound candidates were also screened from the GEO “Drug Perturbations from GEO up” and “Drug Perturbations from GEO down” categories, along with available evidence for possible adverse effects (Table IX; see the supplementary material for the full list).

**TABLE IX:** Drugs Identified in “drug Perturbations From GEO up/down” in Enrichr for the 134 Genes Selected by TD-Based Unsupervised FE

Term	Overlap	P-value	Adjusted P-value
Drug Perturbations from GEO up			
coenzyme Q10 5 281 915 mouse GSE15129 sample 3464	64/302	$1.32\times 10^{-81}$	$1.20\times 10^{-78}$
coenzyme Q10 5 281 915 mouse GSE15129 sample 3456	63/396	$1.05\times 10^{-71}$	$4.78\times 10^{-69}$
captopril DB01197 mouse GSE19286 sample 2689	47/134	$1.76\times 10^{-70}$	$5.33\times 10^{-68}$
ubiquinol 9 962 735 mouse GSE15129 sample 3463	60/346	$2.28\times 10^{-70}$	$5.15\times 10^{-68}$
N-METHYLFORMAMIDE 31 254 rat GSE5509 sample 3570	56/283	$8.87\times 10^{-69}$	$1.61\times 10^{-66}$
1-Naphthyl isothiocyanate 11 080 rat GSE5509 sample 3568	56/301	$3.80\times 10^{-67}$	$5.73\times 10^{-65}$
fenretinide 5 288 209 rat GSE3952 sample 3561	59/397	$6.98\times 10^{-65}$	$9.04\times 10^{-63}$
coenzyme Q10 5 281 915 mouse GSE15129 sample 3462	50/257	$1.49\times 10^{-60}$	$1.69\times 10^{-58}$
bexarotene DB00307 human GSE6914 sample 2680	43/147	$3.03\times 10^{-60}$	$3.05\times 10^{-58}$
FENRETINIDE 5 288 209 rat GSE3952 sample 3563	52/345	$5.99\times 10^{-57}$	$5.42\times 10^{-55}$
Drug Perturbations from GEO up			
pioglitazone DB01132 rat GSE21329 sample 2841	56/321	$1.85 \times 10^{-65}$	$1.67 \times 10^{-62}$
quercetin DB04216 mouse GSE38067 sample 3441	59/486	$1.81 \times 10^{-59}$	$8.19 \times 10^{-57}$
ubiquinol 9 962 735 mouse GSE15129 sample 3461	53/349	$2.64 \times 10^{-58}$	$7.95 \times 10^{-56}$
fenretinide 5 288 209 rat GSE3952 sample 3559	56/440	$2.31 \times 10^{-5}$7	$5.21 \times 10^{-55}$
decitabine 451 668 mouse GSE4768 sample 3108	45/226	$1.06 \times 10^{-54}$	$1.91 \times 10^{-52}$
troglitazone DB00197 rat GSE21329 sample 2832	50/355	$4.51 \times 10^{-53}$	$6.79 \times 10^{-51}$
adenosine triphosphate 5957 human GSE30903 sample 3219	49/341	$2.16 \times 10^{-52}$	$2.78 \times 10^{-50}$
alitretinoin DB00523 rat GSE3952 sample 2673	53/483	$1.54 \times 10^{-50}$	$1.74 \times 10^{-48}$
bexarotene 82 146 rat GSE3952 sample 3560	48/361	$1.42 \times 10^{-49}$	$1.43 \times 10^{-47}$
HYPOCHLOROUS ACID 24 341 human GSE11630 sample 3201	41/221	$2.67 \times 10^{-48}$	$2.41 \times 10^{-46}$
streptozocin DB00428 mouse GSE38067 sample 3439	44/287	$4.40 \times 10^{-48}$	$3.61 \times 10^{-46}$
rosiglitazone DB00412 mouse GSE35011 sample 2813	44/290	$7.13 \times 10^{-48}$	$5.36 \times 10^{-46}$
motexafin gadolinium (4 h) DB05428 human GSE2189 sample 3125	43/302	$1.70 \times 10^{-45}$	$1.18\times 10^{-43}$

Drugs associated with upregulated genes that overlapped with the 134 genes selected by TD-based unsupervised FE are considered to be more likely to cause adverse effects, since they will enhance the expression of genes altered by SARS-CoV infection. Captoprilis is an angiotensin-converting enzyme (ACE) inhibitor, which is known to activate ACE2 that is the receptor that SARS-CoV-2 uses to infect human cells [22], suggesting that this drug might have negative effects for COVID-19 therapy. Coenzyme Q10, which frequently emerged in Table IX, has been reported to accelerate virus infection [23], which could therefore also have negative effects for COVID-19 therapy. Fenretinide is known to effectively inhibit HIV infection [24], and therefore might be a promising drug candidate for SARS-CoV-2 even though it was listed in the “Drug Perturbations from GEO up” category.

In contrast to the drugs in the above list, those associated with downregulated genes that overlapped with the 134 genes selected by TD-based unsupervised FE are considered to be able to effectively suppress SARS-CoV-2 infection, since they will inhibit the expression of genes altered by SARS-CoV infection. Pioglitazone was also included in the list of candidate compounds for SARS-CoV-2 screened by an *in silico* method [25]. Quercetin was reported to inhibit the cell entry of SARS-CoV-2 [26], and was also included in the list of candidate compounds for SARS-CoV-2 screened by an *in silico* method [27]. Fenretinide was also included in the drugs identified as effective compounds in the “Drug perturbations from GEO up” category as described above. Decitabine is one of the drugs used in HIV combination therapy [28]. Troglitazone impedes the oligomerization of sodium taurocholate co-transporting polypeptide and entry of hepatitis B virus into hepatocytes [29], which is a partially double-stranded DNA virus. Finally, motexafin gadolinium was reported to selectively induce apoptosis in HIV-1-infected CD4+ T helper cells [30].

Based on these observations, our strategy appears to be useful to identify potential drug compounds for SARS-CoV-2.

### G. Comparison With *in Silico* Drug Discovery

Finally, we compared the drugs screened out using our approach from the “Drug perturbations from GEO up/down” lists with those screened from two *in silico* drug discovery studies [25], [27]

#### 1) Comparison With Wu et al. [25]

We found multiple hits, which are summarized in Table X.

**TABLE X:** List of *in Silico* Screened drugs [25] Whose Target Genes Were Also Enriched in the 134 Genes Selected by TD-Based Unsupervised FE

Term	Overlap	P-value	Adjusted P-value
Drug Perturbations from GEO up			
doxycycline DB00254 mouse GSE29848 sample 3209	32/267	$3.73\times 10^{-31}$	$3.56\times 10^{-30}$
doxycycline DB00254 human GSE2624 sample 3074	28/175	$6.59\times 10^{-31}$	$6.22\times 10^{-30}$
doxycycline DB00254 human GSE2624 sample 3077	27/209	$3.34\times 10^{-27}$	$2.46\times 10^{-26}$
doxycycline DB00254 human GSE2624 sample 3076	25/272	$1.71\times 10^{-21}$	$8.32\times 10^{-21}$
doxycycline DB00254 mouse GSE29848 sample 3207	22/225	$1.40\times 10^{-19}$	$6.10\times 10^{-19}$
doxycycline DB00254 mouse GSE29848 sample 3208	12/291	$6.24\times 10^{-7}$	$1.40\times 10^{-6}$
ascorbic acid 54 670 067 human GSE11919 sample 3190	8/313	$1.25 \times 10^{-3}$	$2.33 \times 10^{-3}$
isotretinoin DB00982 human GSE10432 sample 2772	8/308	$1.13 \times 10^{-3}$	$2.11 \times 10^{-3}$
pioglitazone DB01132 rat GSE21329 sample 2843	48/400	$2.31\times 10^{-47}$	$7.49\times 10^{-46}$
pioglitazone DB01132 rat GSE21329 sample 2842	42/349	$3.21\times 10^{-41}$	$6.19\times 10^{-40}$
pioglitazone DB01132 rat GSE21329 sample 2842	42/349	$3.21\times 10^{-41}$	$6.19\times 10^{-40}$
pioglitazone DB01132 rat GSE20219 sample 2794	13/292	$8.57\times 10^{-8}$	$2.01\times 10^{-7}$
pioglitazone 4829 mouse GSE1458 sample 2587	11/318	$1.00\times 10^{-5}$	$2.13\times 10^{-5}$
pioglitazone DB01132 mouse GSE32536 sample 2797	7/307	$4.69 \times 10^{-3}$	$8.40 \times 10^{-3}$
pioglitazone DB01132 rat GSE20219 sample 2795	7/330	$6.90 \times 10^{-3}$	$1.22 \times 10^{-2}$
hydrocortisone DB00741 human GSE7890 sample 2751	40/305	$1.01 \times 10^{-40}$	$1.75 \times 10^{-39}$
tibolone 444 008 human GSE12446 sample 3204	15/287	$9.26 \times 10^{-10}$	$2.38 \times 10^{-9}$
Drug Perturbations from GEO down			
doxycycline DB00254 human GSE2624 sample 3075	20/358	$3.39 \times 10^{-13}$	$1.34 \times 10^{-12}$
doxycycline DB00254 mouse GSE29848 sample 3208	17/309	$2.91 \times 10^{-11}$	$1.02 \times 10^{-10}$
doxycycline DB00254 human GSE2624 sample 3077	18/391	$1.37 \times 10^{-10}$	$4.58 \times 10^{-10}$
doxycycline DB00254 mouse GSE29848 sample 3207	17/375	$5.85 \times 10^{-10}$	$1.86 \times 10^{-9}$
doxycycline DB00254 human GSE2624 sample 3074	17/425	$3.88 \times 10^{-9}$	$1.16 \times 10^{-8}$
doxycycline DB00254 mouse GSE29848 sample 3209	9/333	$4.16 \times 10^{-4}$	$8.91 \times 10^{-4}$
ascorbic acid 54 670 067 human GSE11919 sample 3190	19/287	$6.71\times 10^{-14}$	$2.75\times 10^{-13}$
Ascorbic acid 54 670 067 mouse GSE37676 sample 3132	15/306	$2.23\times 10^{-9}$	$6.89\times 10^{-9}$
pioglitazone DB01132 rat GSE21329 sample 2841	56/321	$1.85\times 10^{-65}$	$1.67\times 10^{-62}$
pioglitazone 4829 mouse GSE1458 sample 2587	28/282	$5.89\times 10^{-25}$	$5.26\times 10^{-24}$
pioglitazone DB01132 rat GSE20219 sample 2794	25/308	$3.62\times 10^{-20}$	$2.25\times 10^{-19}$
pioglitazone DB01132 rat GSE20219 sample 2795	21/270	$1.12\times 10^{-16}$	$5.41\times 10^{-16}$
pioglitazone DB01132 human GSE8157 sample 2796	12/269	$2.70\times 10^{-7}$	$7.17\times 10^{-7}$
tibolone 444 008 human GSE12446 sample 3204	35/313	$4.90 \times 10^{-33}$	$8.68 \times 10^{-32}$

The main drugs identified included doxycycline, ascorbic acid, isotretinoin, pioglitazone, cortisone, and tibolone.

Wu *et al.* [25] identified 29 potential PLpro inhibitors, 27 potential 3CLpro inhibitors, and 20 potential RdRp inhibitors from the ZINC drug database, and identified 13 potential PLpro inhibitors, 26 potential 3Clpro inhibitors, and 20 Potential RdRp inhibitors from their in-house natural product database. Doxycycline was among both the potential PLpro and 3CLpro inhibitors; ascorbic acid and isotretinoin were among the potential PLpro inhibitors; pioglitazone was among the potential 3CLpro inhibitors; and cortisone and tibolone were included in the potential RdRp inhibitors from the ZINC drug database. These multiple hits also further support the suitability of our strategy.

#### 2) Comparison With Ubani *et al.* [27]

Ubani *et al.* [27] screened a library of 22 phytochemicals with antiviral activity obtained from the PubChem database for activity against the spike envelope glycoprotein and main protease of SARS-CoV-2. Among these, we found only one hit that overlapped with our screened out drugs, which was quercetin (Table XI).

**TABLE XI:** List of *in Silico* Screened drugs [27] Whose Target Genes are Also Enriched in the 134 Genes Selected by TD Based Unsupervised FE

Term	Overlap	P-value	Adjusted P-value
Drug Perturbations from GEO up			
quercetin DB04216 mouse GSE38141 sample 3435	33/280	$6.85 \times 10^{-32}$	$6.74 \times 10^{-31}$
quercetin DB04216 mouse GSE38136 sample 3438	31/254	$1.99 \times 10^{-30}$	$1.80 \times 10^{-29}$
quercetin DB04216 mouse GSE38136 sample 3437	37/472	$2.85 \times 10^{-29}$	$2.37 \times 10^{-28}$
quercetin 5 280 343 rat GSE7479 sample 3409	33/394	$5.47 \times 10^{-27}$	$3.97 \times 10^{-26}$
quercetin DB04216 mouse GSE38136 sample 3436	30/297	$5.97 \times 10^{-27}$	$4.29 \times 10^{-26}$
quercetin DB04216 mouse GSE38067 sample 3440	26/227	$8.02 \times 10^{-25}$	$4.88 \times 10^{-24}$
quercetin 5 280 343 human GSE7259 sample 3416	29/327	$1.99 \times 10^{-24}$	$1.17 \times 10^{-23}$
quercetin DB04216 mouse GSE38067 sample 3441	20/114	$4.34 \times 10^{-23}$	$2.37 \times 10^{-22}$
quercetin 5 280 343 human GSE13899 sample 3182	16/307	$2.56 \times 10^{-10}$	$6.85 \times 10^{-10}$
quercetin DB04216 mouse GSE4262 sample 3428	14/360	$1.42 \times 10^{-7}$	$3.29 \times 10^{-7}$
quercetin DB04216 mouse GSE4262 sample 3429	9/229	$2.44 \times 10^{-5}$	$5.14 \times 10^{-5}$
quercetin 5 280 343 human GSE7259 sample 3415	9/336	$4.44 \times 10^{-4}$	$8.58 \times 10^{-4}$
quercetin DB04216 mouse GSE4262 sample 3433	8/323	$1.52 \times 10^{-3}$	$2.82 \times 10^{-3}$
quercetin DB04216 mouse GSE4262 sample 3434	8/324	$1.55 \times 10^{-3}$	$2.87 \times 10^{-3}$
quercetin DB04216 mouse GSE4262 sample 3431	7/252	$1.56 \times 10^{-3}$	$2.89 \times 10^{-3}$
quercetin DB04216 mouse GSE4262 sample 3427	8/360	$2.97 \times 10^{-3}$	$5.40 \times 10^{-3}$
quercetin DB04216 mouse GSE4262 sample 3432	5/254	$2.83 \times 10^{-3}$	$4.76 \times 10^{-3}$
Drug Perturbations from GEO down			
quercetin DB04216 mouse GSE38067 sample 3441	59/486	$1.81 \times 10^{-59}$	$8.19 \times 10^{-57}$
quercetin DB04216 mouse GSE38136 sample 3437	26/128	$1.33 \times 10^{-3}$1	$2.00 \times 10^{-30}$
quercetin 5 280 343 human GSE7259 sample 3415	29/264	$4.05 \times 10^{-27}$	$4.57 \times 10^{-26}$
quercetin DB04216 mouse GSE38136 sample 3436	30/303	$1.09 \times 10^{-26}$	$1.17 \times 10^{-25}$
quercetin 5 280 343 human GSE13899 sample 3182	26/293	$6.10 \times 10^{-22}$	$4.55 \times 10^{-21}$
quercetin DB04216 mouse GSE38067 sample 3440	28/373	$1.31 \times 10^{-21}$	$9.44 \times 10^{-21}$
quercetin DB04216 mouse GSE38136 sample 3438	27/346	$2.71 \times 10^{-21}$	$1.91 \times 10^{-20}$
quercetin DB04216 mouse GSE38141 sample 3435	22/320	$2.68 \times 10^{-16}$	$1.26 \times 10^{-15}$
quercetin 5 280 343 human GSE7259 sample 3416	18/273	$3.44 \times 10^{-13}$	$1.36 \times 10^{-12}$
quercetin 5 280 343 rat GSE7479 sample 3409	14/206	$1.12 \times 10^{-10}$	$3.81 \times 10^{-10}$
quercetin DB04216 mouse GSE4262 sample 3431	11/348	$2.31 \times 10^{-5}$	$5.47 \times 10^{-5}$
quercetin DB04216 mouse GSE4262 sample 3427	5/240	$2.28 \times 10^{-3}$	$4.21 \times 10^{-3}$

## IV. Discussion and Conclusion

In this paper, we present a novel evaluation method to identify drugs that could be used to effectively treat COVID-19. We applied a TD-based unsupervised FE method to select genes with altered expression caused by MHV infection in mice. Although the dataset analyzed for this study was not based on SARS-CoV-2 infection, the 134 genes selected by TD-based unsupervised FE can still be considered useful for gaining a better understanding of the infectious mechanism of SARS-CoV-2 for several reasons. First, the 134 genes selected were enriched in general RNA virus proteins that play important roles during infectious processes. This suggests that the infectious mechanism represented by the 134 genes in the mouse model is also applicable to SARS-CoV-2 infection. In fact, these genes were also enriched in processes related to scavenger receptor activity, which might reflect the critical role of TMPRSS2 activity in SARS-CoV-2 replication, suggesting a potential therapeutic target.

Following these achievements, we tried to identify potential drug candidate compounds that could influence the 134 selected genes. Among these, we screened out several candidate compounds that are known antiviral drugs, including those that were screened out as drug candidate compounds for SARS-CoV-2 using *in silico* methods.

**Fig. 5.**
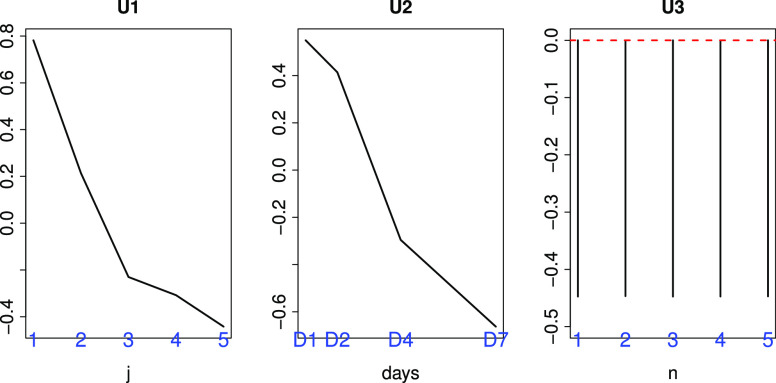
Singular value vectors obtained by the HOSVD algorithm applied to GSE33266 (Table II). U1:$u_{2j}$, U2:$u_{\text{2}\,k}$, and U3:$u_{1n}$. See Materials and Methods for the meanings of $j, k$ and $n$.

**Fig. 6.**
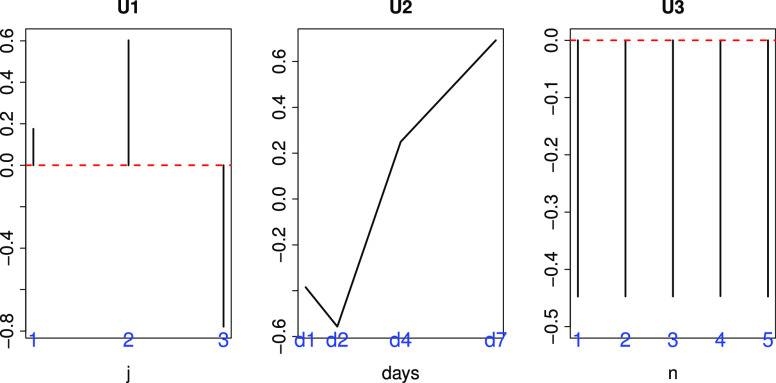
Singular value vectors obtained by the HOSVD algorithm applied to GSE50000 (Table II). U1:$u_{2j}$, U2:$u_{\text{3}\,k}$, and U3:$u_{1n}$. See Materials and Methods for the meanings of $j, k$ and $n$.

The question arises whether MHV is a suitable model system for SARS-CoV-2 infection, since there are more datasets available for SARS-CoV infection in mouse lung. In order to evaluate the suitability of MHV as a model of the SARS-CoV-2 infectious process, we also performed additional analyses using two SARS-CoV infectious processes in mouse lung (see Materials and Methods). As can be seen in Figs. 5 and 6, $\ell _1=2$ was selected as singular value vector $u_{\ell _1 j}$ with monotonic dependence upon $j$, $\ell _2=2$ was selected as a singular value vector $u_{\ell _2\,k}$ with monotonic dependence upon $k$, and $\ell _3=1$ was selected as a singular value vector $u_{\ell _3 n}$ with constant values regardless of $n$ for GSE33266, while $\ell _1=2$ was selected as a singular value vector $u_{\ell _1 j}$ with distinct values between mock ($j=3$) and infectious samples ($j=1,2$), $\ell _2=3$ was selected as a singular value vector $u_{\ell _2\,k}$ with monotonic dependence upon $k$, and $\ell _3=1$ was selected as a singular value vector $u_{\ell _3 n}$ with constant values regardless of $n$ for GSE50000. Then $\ell _4=2,3$ and $\ell _4=1$ were selected for GSE33266 and GSE50000, respectively, as those associated with the larger absolute values of $G(\ell _1 \ell _2 \ell _3 \ell _4)$ given $\ell _1, \ell _2, \ell _3$. $P$-values, $P_i$, were attributed to gene $i$ using a cumulative $\chi ^2$ distribution, $P_{\chi ^2} [>x]$, as described in the Materials and Methods; 569 gene symbols associated with selected genes were selected for GSE33266 and 475 were selected for GSE50000 (see Supplementary Material).

**Fig. 7.**
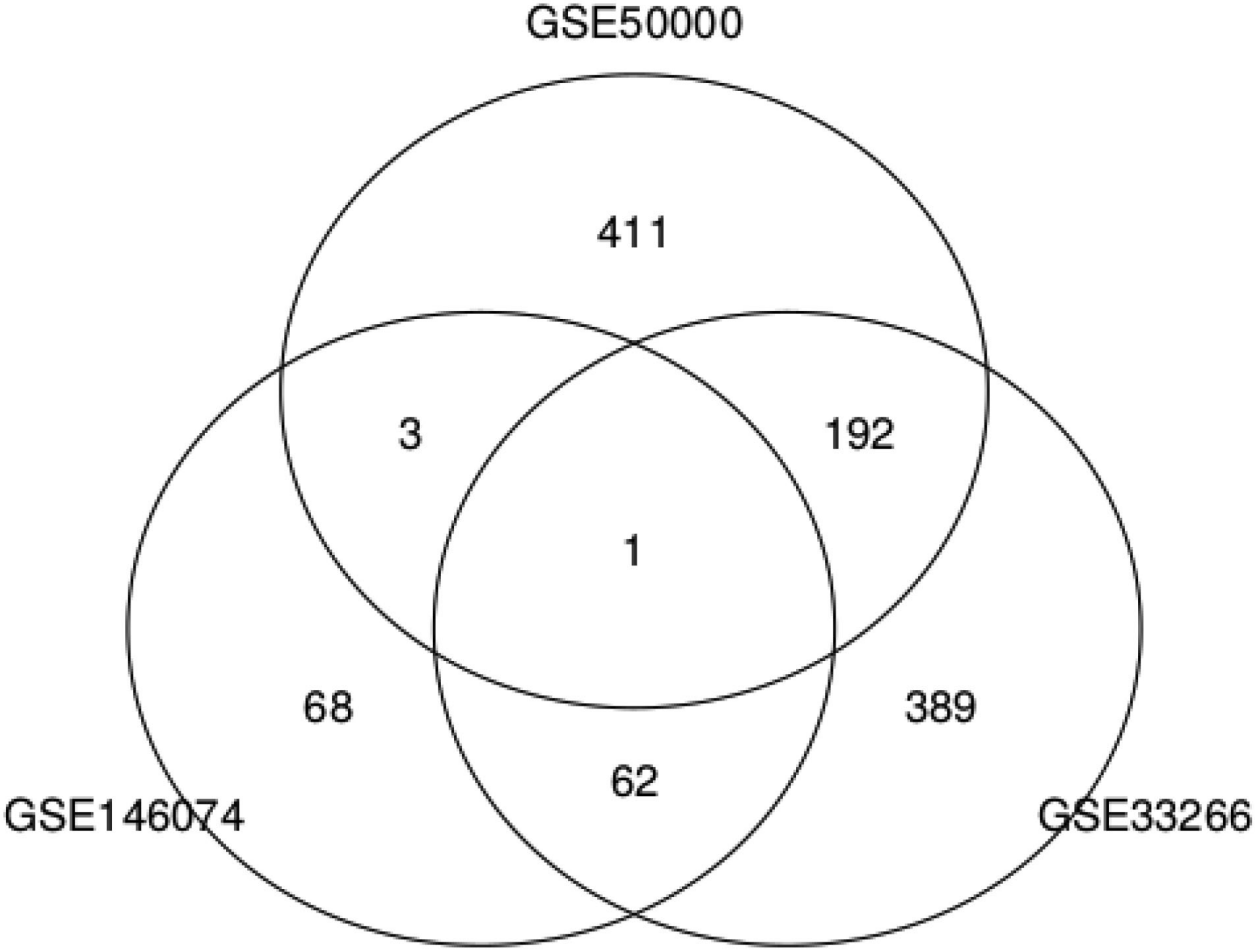
Venn diagrams of 134 genes selected by TD bases unsupervised FE using GSE146074, 569 genes selected by TD bases unsupervised FE using GSE33266, and 475 genes selected by TD bases unsupervised FE using GSE50000.

Fig. 7 shows the Venn diagram of these two sets of genes and 134 genes selected by TD based unsupervised FE using GSE146074. Although there are some overlaps, the majority of genes are not shared among these three gene sets.

These two sets of genes were uploaded to Enrichr and checked for significant overlap with coronavirus PPI genes (Table XII).Two sets of selected genes significantly overlap with genes that are believed to interact with SARS-CoV proteins, in spite of the fact that these two gene sets are not highly coincident with the 134 genes selected by the TD based unsupervised FE using GSE146074 (Fig. 7). TD based unsupervised FE therefore has the ability to predict PPI using gene expression profiles, no matter which data sets among GSE146074, GSE33266, and GSE50000 are used. These findings also suggest that the results shown in Table VI are unlikely to be accidental, but provide evidence that the genes selected by TD based unsupervised FE are those interacting with SARS-CoV proteins during infectious processes.

**TABLE XII:** SARS-CoV-Related Virus PPI in Enrichr

Term	Overlap	P-value	Adjusted P-value
GSE33266			
SARS coronavirus excised_polyprotein 1..4369 (gene: orf1ab)	16/194	$3.21\times 10^{-6}$	$1.54\times 10^{-3}$
SARS coronavirus P2 full_polyprotein 1..4382	16/198	$4.18\times 10^{-6}$	$1.65\times 10^{-3}$
SARS coronavirus nsp9-pp1a/pp1ab (gene: orf1ab)	5/13	$4.26\times 10^{-6}$	$1.59\times 10^{-3}$
SARS coronavirus 3C-like proteinase (gene: orf1ab)	5/19	$3.47\times 10^{-5}$	$4.09\times 10^{-3}$
SARS coronavirus nsp8-pp1a/pp1ab (gene: orf1ab)	7/45	$3.67\times 10^{-5}$	$4.18\times 10^{-3}$
SARS coronavirus 2-O-ribose methyltransferase (2-o-MT) (gene: orf1ab)	4/11	$5.42\times 10^{-5}$	$4.73\times 10^{-3}$
SARS coronavirus nsp7-pp1a/pp1ab (gene: orf1ab)	6/36	$8.97\times 10^{-5}$	$6.77\times 10^{-3}$
SARS coronavirus endoRNAse (gene: orf1ab)	3/6	$1.71\times 10^{-4}$	$1.11\times 10^{-2}$
SARS coronavirus nsp4-pp1a/pp1ab (gene: orf1ab)	4/16	$2.75\times 10^{-4}$	$1.61\times 10^{-2}$
SARS coronavirus formerly known as growth-factor-like protein (gene: orf1ab)	4/17	$3.54\times 10^{-4}$	$1.93\times 10^{-2}$
SARS coronavirus Tor2 replicase 1AB	9/108	$4.26\times 10^{-4}$	$2.22\times 10^{-2}$
SARS coronavirus P2 full_polyprotein 1..7073	9/109	$4.56\times 10^{-4}$	$2.32\times 10^{-2}$
SARS coronavirus RNA-dependent RNA polymerase (gene: orf1ab)	3/9	$6.84\times 10^{-4}$	$2.94\times 10^{-2}$
SARS coronavirus leader protein (gene: orf1ab)	4/20	$6.86\times 10^{-4}$	$2.92\times 10^{-2}$
SARS coronavirus nsp3-pp1a/pp1ab (gene: orf1ab)	9/118	$8.12\times 10^{-4}$	$3.22\times 10^{-2}$
GSE50000			
SARS coronavirus 3C-like proteinase (gene: orf1ab)	4/19	$4.21\times 10^{-4}$	$1.62\times 10^{-2}$
SARS coronavirus hypothetical protein sars7a	5/38	$7.75\times 10^{-4}$	$1.94\times 10^{-2}$
SARS coronavirus P2 hypothetical protein sars7a	5/38	$7.75\times 10^{-4}$	$1.94\times 10^{-2}$
SARS coronavirus Tor2 Orf8	5/38	$7.75\times 10^{-4}$	$1.93\times 10^{-2}$
SARS coronavirus 2-O-ribose methyltransferase (2-o-MT) (gene: orf1ab)	3/11	$1.05\times 10^{-3}$	$2.12\times 10^{-2}$
SARS coronavirus nsp9-pp1a/pp1ab (gene: orf1ab)	3/13	$1.77\times 10^{-3}$	$2.43\times 10^{-2}$
SARS coronavirus nsp13-pp1ab (ZD, NTPase/HEL; RNA (gene: orf1ab)	3/14	$2.22\times 10^{-3}$	$2.49\times 10^{-2}$
SARS coronavirus P2 spike glycoprotein precursor	6/71	$2.48\times 10^{-3}$	$2.69\times 10^{-2}$
SARS coronavirus E2 glycoprotein precursor (gene: S)	6/72	$2.66\times 10^{-3}$	$2.78\times 10^{-2}$
SARS coronavirus Tor2 spike glycoprotein	6/72	$2.66\times 10^{-3}$	$2.78\times 10^{-2}$
SARS coronavirus nsp4-pp1a/pp1ab (gene: orf1ab)	3/16	$3.31\times 10^{-3}$	$2.94\times 10^{-2}$
SARS coronavirus nsp3-pp1a/pp1ab (gene: orf1ab)	7/118	$7.99\times 10^{-3}$	$3.74\times 10^{-2}$

Another possible concern is that the studies are not based upon direct investigation of SARS-CoV-2, but are based upon a closely related virus. This limitation suggests that these results might not be applicable to SARS-CoV-2. In order to address this point, we compared the 134 genes (Table V) with genes reported to interact with SARS-CoV-2 proteins [31] (Table XIII).One hundred and thirty-four genes significantly overlap with human genes reported to interact with SARS-CoV-2 proteins. TD based unsupervised FE therefore appears to have the ability to predict PPI, even when only gene expression profiles from a related virus are available. Especially, it is remarkable that the present study could outperform the inference in the previous study [8] (asterisked ones in Table XIII) where SARS-CoV-2 infected human lung cell lines are investigated. This is possibly because of the superiority of *in vivo* study toward *in vitro* study in spite of the usage of different species. Our proposed method, TD based unsupervised FE, can make use of the data set taken from different species infected by related but not exactly same virus whereas other methods could not (see below). Since it is unrealistic to intentionally infect human subject SARS-CoV-2, it is important to have the methodology that can make use of data set taken from other species than human.

**TABLE XIII:** Coincidence Between 134 Genes and Human Genes Reported to Interact With SARS-CoV-2 Proteins [31]. Asterisked Ones are the Cases That Outperformed the Inference in Previous Study [8] That Investigated Gene Expression Profiles of Human Lung Cell Lines Infected by SARS-CoV-2

SARS-CoV-2 proteins	P values	Odds Ratio
SARS-CoV2 E	$1.59\times 10^{-41}$ (*)	20.2 (*)
SARS-CoV2 M	$1.53\times 10^{-34}$ (*)	13.7 (*)
SARS-CoV2 N	$1.69\times 10^{-51}$ (*)	31.8 (*)
SARS-CoV2 nsp1	$5.56\times 10^{-33}$ (*)	19.8 (*)
SARS-CoV2 nsp10	$4.16\times 10^{-32}$ (*)	23.0 (*)
SARS-CoV2 nsp11	$3.59\times 10^{-41}$ (*)	19.1 (*)
SARS-CoV2 nsp12	$4.29\times 10^{-31}$ (*)	18.3 (*)
SARS-CoV2 nsp13	$7.26\times 10^{-42}$ (*)	18.6 (*)
SARS-CoV2 nsp14	$1.64\times 10^{-32}$ (*)	22.3 (*)
SARS-CoV2 nsp15	$3.72\times 10^{-26}$ (*)	16.4 (*)
SARS-CoV2 nsp2	$5.25\times 10^{-48}$ (*)	22.7 (*)
SARS-CoV2 nsp4	$2.18\times 10^{-35}$ (*)	16.0 (*)
SARS-CoV2 nsp5	$4.30\times 10^{-40}$ (*)	25.5 (*)
SARS-CoV2 nsp5_C145 A	$8.22\times 10^{-31}$ (*)	25.1 (*)
SARS-CoV2 nsp6	$1.52\times 10^{-36}$ (*)	16.0 (*)
SARS-CoV2 nsp7	$6.93\times 10^{-31}$ (*)	14.7 (*)
SARS-CoV2 nsp8	$3.51\times 10^{-44}$ (*)	19.6 (*)
SARS-CoV2 nsp9	$2.62\times 10^{-42}$ (*)	23.3 (*)
SARS-CoV2 orf10	$2.67\times 10^{-46}$ (*)	22.3 (*)
SARS-CoV2 orf3a	$8.57\times 10^{-44}$ (*)	19.8 (*)
SARS-CoV2 orf3b	$3.79\times 10^{-47}$ (*)	24.8 (*)
SARS-CoV2 orf6	$1.51\times 10^{-42}$ (*)	21.9 (*)
SARS-CoV2 orf7a	$6.28\times 10^{-34}$ (*)	15.3 (*)
SARS-CoV2 orf8	$1.90\times 10^{-33}$ (*)	14.0 (*)
SARS-CoV2 orf9b	$3.16\times 10^{-42}$ (*)	22.3 (*)
SARS-CoV2 orf9c	$8.73\times 10^{-38}$ (*)	13.9 (*)

Finally, we compared identified drugs (“Drug Pert GEO up/down” and “DrugMarix“ in the Supplementary Material) with those reported as possible drugs against SARS-CoV-2 [32]. Among the 142 drugs identified by Zhou *et al.* [32], as many as 25 drugs were found to significantly affect 134 genes in at least one experiment within either DrugMatrix, or GEO, using Enrichr with adjusted $P$-values of less than 0.05 (Table XIV). Thus, our suggestions for drug repositioning are also supported.

**TABLE XIV:** Number of Experiments Associated With Adjusted $P$-Values in Various Enrichr Categories for the Drugs Identified in Another study [32]

	GEO up	GEO down	DrugMatrix
Methotrexate	2	1	35
Fluorouracil	4	5	3
Testosterone	1		12
Stanolone	2	1	
Menadione		1	
Hydrocortisone		1	20
Mestranol			6
Hexestrol			4
Mercaptopurine			15
Paroxetine			8
Vinblastine			16
Phenylbutazone			3
Naloxone			6
Hydralazine			11
Vinorelbine			10
Carvedilol			16
Colchicine			12
Amitriptyline			12
Epinephrine			12
Dactinomycin			6
Melatonin			8
Methyltestosterone			6
Omeprazole			19
Oxymetholone			6
Progesterone			20

Since it is unlikely that this level of agreement is purely accidental, the drugs identified in the present study can be useful candidates for further evaluation for COVID-19 therapy. This work therefore provides a foundation for further research pertaining to utilizing advanced learning concepts to analyze COVID-19 infectious disease.

Final concerns to be addressed might be the comparison with methods other than TD based unsupervised FE applied to synthetic data sets. When considering the synthetic data set, categorical regression, eq. (10), outperformed TD based unsupervised FE. Although eq. (9) cannot be better than TD based unsupervised FE (Table III), its performance is still comparable. If these two more easily understood methods are better than or comparable to TD based unsupervised FE, TD based unsupervised FE, which is more difficult to interpret, is useless. In order to check this point, we applied categorical regression and eq. (9), which were modified as

\begin{equation*}
x_{ijkmnp} = \alpha _{ijkm} + \gamma ^{\prime \prime }_i \tag{13}
\end{equation*}

and

\begin{equation*}
x_{ijkmnp} = \alpha ^{\prime }_i a_j b_k c_m +\gamma ^{\prime }_i \tag{14}
\end{equation*}

where $a_i=i, b_k=k, c_m=m$, to the present set (GSE146074). Then $i$s associated with adjusted $P$-values less than 0.01 were selected. There are too many genes that passed this screening to evaluate: 25 609 and 20 217, respectively. This observation suggests that these two methods lack the ability to screen for a limited number of genes that are likely to interact with SARS-CoV-2 proteins, since only a limited number of human genes will interact with SARS-CoV-2 proteins ($2 \times 10^4$ are as many as all human protein coding genes). Although this finding is enough reason to reject the use of these two methods in favor of TD based unsupervised FE if only the top ranked genes are selected, it might be possible to identify a limited number of genes that significantly overlap with genes reported to interact with SARS-CoV-2 proteins. We selected the 134 top ranked genes using the $P$-values produced by these two methods and uploaded them to Enrichr. There were no SARS-CoV-2 proteins that significantly interact with these 134 genes. Since a significant interaction with SARS-CoV-2 proteins is the primary requirement for genes to be used to screen candidate drug compounds, these two methods appear to be inadequate for the present purpose.

If we employ more complicated and sophisticated methods to select genes, it might be possible to identify a limited number of genes that significantly interact with SARS-CoV-2 proteins. However, TD based unsupervised FE is simple and rapid (see the CPU time in Table III) enough to achieve the present purpose, and does so with acceptable accuracy.

Finally, we would like to discuss why we did not use SARS-CoV-MA15 data. This is simply because we could not get any significant overlaps with human proteins supposed to be interact with SARS-CoV proteins. This is possibly because SARS-CoV-MA15 is believed to be adapted to mouse infection processes while we checked human proteins. Thus, even if organism used is not human but mouse, MHV is suitable model for human SARS-CoV-2 infection and our method has ability to distinguish between MHV and SARS-CoV-15 where the former remains an effective model of human SARS-CoV-2 infectious process as Pfaender *et al.* [6] correctly assumed while the latter is not.

One might wonder why we have employed one specific algorithm, HOSVD, among those developed to apply TD to tenors. Although it was fully described in the recently published book [7], we outline it here very briefly.

• Although CP decomposition [7] is more popular implementation, it cannot give us suitable solution when the number of features are much larger than that of samples as the problems dealt in this study because of its heavily dependence upon initial values [7].
• There are other implementations to derive Tucker decomposition than HOSVD, other methods cannot give us suitable solution because other methods require the optimization within the limited number of given singular value vectors. Since HOSVD does not perform optimization and singular value vectors can be obtained independent of the number of singular value vectors to be computed, HOSVD does not destroy important singular value vectors with small contribution; since the number of genes considers is as small as a few hundreds, which is less than 1 % of total number of genes $\simeq 10^4$, we cannot ignore singular value vectors with small contributions that might reflect the property of a few hundred genes.
• Not all TD cannot give us weight to evaluate the relations between singular value vectors attributed to distinct instances. Although HOSVD can give us $G$ that can evaluate coincidence between $u_{\ell _t}, 1 \leq t \leq 6$, for example tensor train decomposition [7] cannot give us something that corresponds to $G$. Thus we cannot know which singular value vectors attributed to gene should be used for selecting genes based upon the evaluation other singular value vectors attributed to, e.g., samples.

Because of these reasons, we have specifically employed HOSVD.

In the book [7], we carried out decompositions of data modeled as three-mode tensor and five-mode tensor in addition to others, which were different from this study as we consider a decomposition of different application and data modeled as six-mode tensor $(x_{ijkmnp} \in \mathbb {R}^{N \times 2 \times 2 \times 3 \times 3 \times 2})$.
